# How metals fuel fungal virulence, yet promote anti-fungal immunity

**DOI:** 10.1242/dmm.050393

**Published:** 2023-10-31

**Authors:** Alanoud Alselami, Rebecca A. Drummond

**Affiliations:** Institute of Immunology and Immunotherapy, University of Birmingham, Birmingham, B15 2TT, UK

**Keywords:** Copper, Fungal immunology, Iron, Macrophage, Nutritional immunity

## Abstract

Invasive fungal infections represent a significant global health problem, and present several clinical challenges, including limited treatment options, increasing rates of antifungal drug resistance and compounding comorbidities in affected patients. Metals, such as copper, iron and zinc, are critical for various biological and cellular processes across phyla. In mammals, these metals are important determinants of immune responses, but pathogenic microbes, including fungi, also require access to these metals to fuel their own growth and drive expression of major virulence traits. Therefore, host immune cells have developed strategies to either restrict access to metals to induce starvation of invading pathogens or deploy toxic concentrations within phagosomes to cause metal poisoning. In this Review, we describe the mechanisms regulating fungal scavenging and detoxification of copper, iron and zinc and the importance of these mechanisms for virulence and infection. We also outline how these metals are involved in host immune responses and the consequences of metal deficiencies or overloads on how the host controls invasive fungal infections.

## Teaser

**Summary:** Transition metals, such as copper, iron and zinc, are critical determinants of host immune responses to fungal pathogens. Paradoxically, metals may also support fungal virulence.

## Introduction

Fungi cause serious, life-threatening infections and have a significant global impact on human health that is often under-appreciated (Table 1). Approximately one billion people are estimated to be affected by fungal diseases each year, with an estimated 1.5 million deaths caused by invasive or systemic fungal infections globally (Rodrigues and Nosanchuk, 2020). The World Health Organization released its first ever fungal pathogen priority list in 2022, which urged for more research and better health policy to tackle the rising threat that these diseases present, particularly in low- to middle-income countries (Fisher and Denning, 2023). Cryptococcal meningitis causes the majority of fungal-related deaths, followed by aspergillosis and candidiasis (Firacative, 2020; Rajasingham et al., 2022). These diseases are associated with specific risk factors, but typically affect people with compromised immune systems caused by organ transplantation, chemotherapy and advanced HIV infection leading to AIDS (Lionakis et al., 2023). An outline of how the immune system protects against fungal infection is provided in Box 1.

**Box 1. Overview of antifungal immune responses**

Fungi are recognized by innate immune cells via extracellular and intracellular pattern recognition receptors (PRRs), including the C-type lectin receptors (CLRs), Toll-like receptors (TLRs) and NOD-like receptors (NLRs) (Lionakis et al., 2023). CLRs, such as dectin-1 (CLEC7A) and dectin-2 (CLEC6A), are the main receptor group mediating antifungal recognition (Plato et al., 2015), and loss of their shared signalling adaptor molecule, CARD9 (Box 2), results in heightened susceptibility to fungal infections (Drummond et al., 2018; Gross et al., 2006). CLRs typically bind to components of the fungal cell wall, including the carbohydrates β-glucan and α-mannose, and subsequently activate intracellular signalling cascades via direct (dectin-1) or indirect (dectin-2) binding to ITAM-like motifs. This activates Syk kinase (Box 2) and a trimolecular complex consisting of BCL10, CARD9 and MALT1 (Box 2). Additional Syk- and CARD9-independent pathways have been described downstream of CLRs following fungal recognition (Lionakis et al., 2017). TLRs are also important for antifungal recognition, with some members binding to cell wall components (e.g. TLR2 and TLR6), whereas intracellularly-expressed TLRs recognise fungal DNA (e.g. TLR9). These TLRs activate intracellular defence mechanisms (Bercusson et al., 2017), but it is important to note that loss of the shared TLR signalling adaptor, MyD88 (Box 2), does not promote increased susceptibility to fungal infections in humans (von Bernuth et al., 2008).

PRR signalling following innate fungal recognition leads to several cellular immune responses, such as phagocytosis, production of reactive oxygen species (ROS) and the release of cytokines. This occurs through downstream activation of transcription factors, including NFκB (Box 2), which regulate the expression of pro-inflammatory cytokines, such as IL-6, TNFα and IL-12. CARD9 also activates extracellular signal-regulated kinases (ERKs) (Box 2) to further enhance cytokine production via RASGRF1 and H-Ras signalling (Jia et al., 2014). These pro-inflammatory cytokines are important for the recruitment and differentiation of more innate and adaptive immune cells.

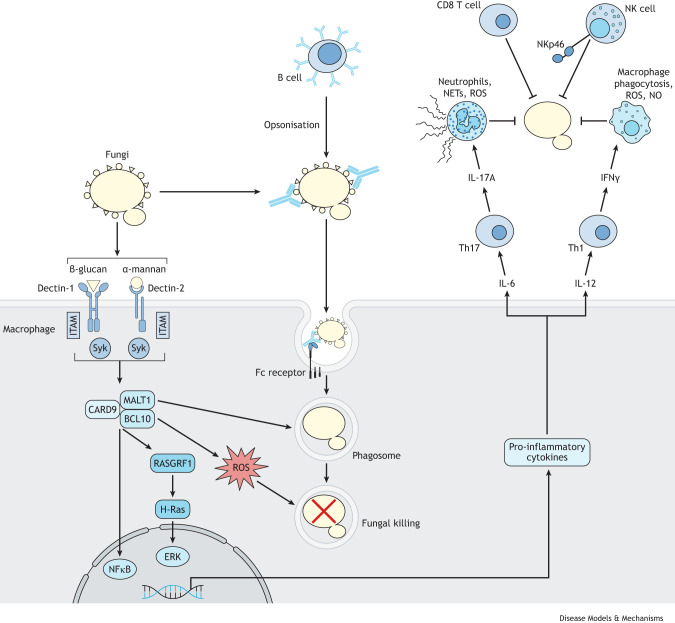

Innate immune cell responses are critical for protection against most invasive fungal infections. Indeed, myeloid cell dysfunction promotes susceptibility to infections with *Candida*, *Aspergillus* and *Cryptococcus* species in humans (Lionakis et al., 2023; Jirapongsananuruk et al., 2012). Macrophages contribute towards fungal killing and clearance primarily through intracellular killing following phagocytosis of fungal cells. Inflammatory macrophages may also kill fungi via ROS. ROS are primarily produced by NADPH oxidase (Box 2) and by mitochondria, and cause extensive damage to the cell walls and membranes of fungi (Gazendam et al., 2016). Patients with mutations in key components of NADPH oxidase, such as in chronic granulomatous disease, are unable to make sufficient ROS, leading to increased susceptibility to life-threatening fungal infections (Leiding and Holland, 2014; Lionakis et al., 2017). Macrophages can also utilize nitrogen-based killing mechanisms via the production of nitric oxide (NO; Box 2) generated by the enzyme iNOS (Box 2) (Orekhov et al., 2019). Macrophages that utilize an alternative arginine metabolic pathway and instead express arginase to produce ornithine and polyamines are typically less capable of killing fungi and may actually enable the development of intracellular infection reservoirs (Dang et al., 2022). This is particularly true for infection with *Cryptococcus* species, where arginase expression in macrophages correlates with fungal proliferation within phagosomes and poor infection control (Dang et al., 2022). For other fungal species, the role of arginase-expressing macrophages is less clear. For example, alveolar macrophages highly express arginase during pulmonary infection with *Aspergillus*, and removal of macrophages with clodronate reduces lung fungal burden (Bhatia et al., 2011). However, the specific role of arginase in macrophage-mediated protection is not clear as MyD88-deficient mice, which are highly susceptible to *Aspergillus* infection, expressed more arginase than wild-type controls (Bhatia et al., 2011). The specific roles of arginase and iNOS and whether their levels correlate with macrophage subsets require further in-depth studies with cell-type-specific knockout mice to bring clarity to how macrophages mediate fungal killing during different infections.

A reduction in circulating neutrophils (neutropenia) in humans is a major risk factor for developing invasive candidiasis (Lionakis et al., 2023; Jirapongsananuruk et al., 2012). One of the primary processes by which neutrophils kill fungi is through the production of ROS (Gazendam et al., 2016). Neutrophils may also kill fungi that are larger than themselves, such as hyphae (Box 2), using extracellular killing mechanisms. NETosis is the process by which neutrophils decondense and release chromatin bound to several antimicrobial factors (e.g. calprotectin, histones) to ‘trap’ fungi and damage their cell walls and membranes externally (Wu et al., 2019).

Adaptive immunity, mediated by lymphocytes, is also essential for eliminating fungal infections. In particular, loss or dysfunction of CD4 T cells in humans promotes a striking susceptibility to cryptococcal meningitis (Williamson et al., 2017). CD4 T cells are also required for defence against mucosal *Candida* infections and invasive infections from the endemic dimorphic fungi (e.g. *Blastomyces*, *Histoplasma*) (Richardson and Moyes, 2015; Lionakis et al., 2023). Following their activation via MHCII-peptide complexes, CD4 T cells produce cytokines that promote fungal killing mechanisms within innate immune cells. For example, type 1 helper (Th1) CD4 T cells produce IFNγ that drives iNOS expression in macrophages, thus enabling efficient clearance of intracellular fungal pathogens, such as *C. neoformans* (Chen et al., 2005)*.* In the mucosa, Th17 CD4 T cells producing IL-17A can enable recruitment of neutrophils and activate expression of antimicrobial peptides to bolster defences against *Candida* colonization and infection at these barrier sites (Conti and Gaffen, 2015; Hernandez-Santos et al., 2013). CD8 T cells and natural killer (NK) cells have both been shown to mediate direct fungal killing by deploying toxic granule contents, such as granzyme and perforin, into fungal cells using receptors, such as NKp46 (or NCR1) (Schmidt et al., 2013). Antibodies produced by B cells are also involved in antifungal immunity, particularly for fungal species that may shield against PRR-mediated recognition (Iseki et al., 1994). For example, *C. neoformans* forms a large polysaccharide capsule that prevents efficient recognition by the CLRs, and antibody-dependent opsonisation (Box 2) is required for efficient phagocytosis and killing of this fungus by macrophages (Ulrich and Ebel, 2020).

**Box 2. Glossary**

**BCL10, CARD9 and MALT1:** signalling adaptor proteins involved in downstream signalling of many members of the C-type lectin receptor (CLR) family, including dectin-1 and dectin-2. BCL10, CARD9 and MALT1 form a trimolecular complex.

**Conidia:** non-mobile fungal spores that replicate by budding at the tips of specialized hyphae called conidiophores.

**Disseminated infection:** clinical term used to describe an infection that has infiltrated multiple organs of the body or entered the bloodstream.

**Diabetic ketoacidosis:** a severe condition that impacts individuals with diabetes, where the body excessively breaks down fat. The liver converts this fat into ketones, a type of fuel, leading to acidic blood conditions.

**ERKs:** signalling kinases activated by H-Ras and RASGRF1, which mediate cellular responses downstream of innate recognition receptors such as CLRs.

**Hyphae:** the filamentous morphological form of fungi, identified as branching tubes that are observed during mycelial or invasive growth.

**iNOS:** one of three enzymes that produce nitric oxide (NO) from the amino acid L-arginine. Its expression is induced in macrophages following stimulation with pro-inflammatory cytokines, such as IFNγ.

**Iron permeases:** fungal and bacterial proteins that are essential for iron transport in iron-depleted environments. Some, like the *Candida albicans* (Ca)FTR1, are essential virulence factors.

**Metallothioniens:** cysteine-rich Golgi membrane proteins involved in metal detoxification.

**MyD88:** a signalling adaptor protein involved in downstream signalling of many members of the Toll-like receptor (TLR) family.

**NADPH oxidase:** a multi-component enzyme found in phagolysosome membranes that generates reactive oxygen species (ROS) to enable microbial killing in phagocytes.

**Necroptosis:** a programmed inflammatory mode of cell death. Unlike in necrosis, cell membrane permeabilisation is tightly regulated via TNFα and the downstream kinase RIPK1.

**NFκB:** a transcription factor that is a master regulator of immune responses, driving expression of multiple pro-inflammatory cytokines including IL-6 and IL-12.

**Nitric oxide (NO):** a toxic nitrogen-derived free radical with anti-microbial properties. It is generated in phagocytes by iNOS.

**Opsonisation:** an immunological mechanism that enhances phagocytosis of pathogens by phagocytes. Antibodies and complement proteins act as opsonins, labelling pathogens for more efficient uptake by phagocytic receptors (e.g. FcγR and CR3).

**Siderophores:** small proteins that are typically secreted and sequester metals.

**Superoxide dismutase:** an enzyme that catalyses the conversion of superoxide radicals (O^-^_2_) into molecular oxygen (O_2_) and hydrogen peroxide (H_2_O_2_). This enzymatic action serves as a cellular defence against ROS.

**Syk:** an intracellular tyrosine kinase participating in the signalling of immunoinflammatory cell drivers, particularly downstream of the CLRs.

**Table 1.**
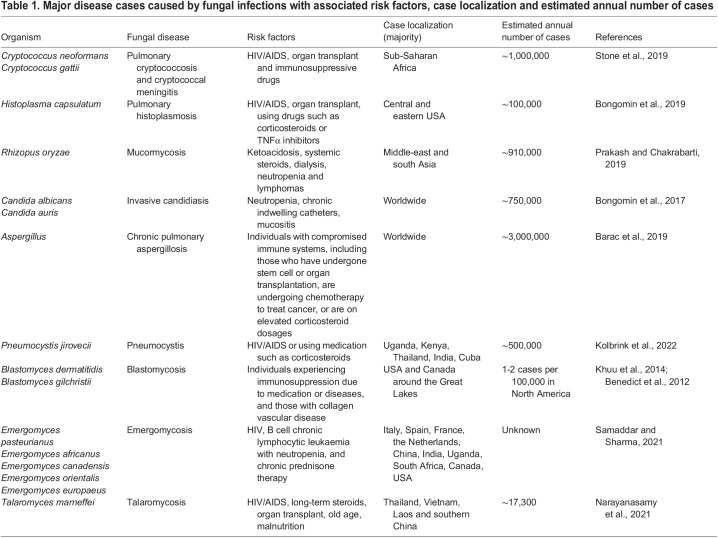
Major disease cases caused by fungal infections with associated risk factors, case localization and estimated annual number of cases

Similar to other pathogenic microbes, fungal morphology and virulence are significantly influenced by metal concentrations in the environment. Metals, such as copper, iron and zinc, are essential elements for several basic cellular processes, such as respiration, energy production and DNA synthesis (Crawford and Wilson, 2015), and are also required by host immune cells to drive immune responses and protect against infection. The host may limit access to metals and other micronutrients from pathogens to restrict their growth, and/or deploy excess concentrations of metals to cause toxicity and mediate antimicrobial killing. These processes are collectively referred to as nutritional immunity (Crawford and Wilson, 2015; Cassat and Skaar, 2013; Murdoch and Skaar, 2022). However, to counteract these strategies, pathogenic microorganisms can initiate metal scavenging or detoxifying mechanisms to protect themselves against starvation and toxicity, many of which have been shown to be critical for survival and virulence (Potrykus et al., 2014). Whether these strategies can be targeted for therapeutic gain remains to be determined, but this would require a greater understanding of how metals fuel fungal infection and/or protect against disease through their role in immune responses.

In this Review, we describe examples of acquisition and detoxification strategies of common essential metals (copper, iron and zinc) by human fungal pathogens and highlight the importance of metal-dependent nutritional immunity in antifungal immune responses. Finally, we discuss how advancing our understanding of these strategies can help develop therapies to target the unacceptably high mortality rates associated with invasive fungal infections.

## Copper

Copper participates in a variety of basic biological processes, including acting as an antioxidant and being an important co-factor for mitochondrial enzymes and respiration. Copper is also used for biosynthesis of neurotransmitters and haem (Lutsenko et al., 2019). Although essential for life, copper is toxic in high concentrations and, as a result, the levels of this metal are under tight homeostatic control. In mammals, high-affinity copper uptake protein 1 (CTR1) mediates copper uptake in cells and tissues, in addition to P-type ATPase copper transporters (ATP7A and ATP7B), which mediate copper internal sequestration and efflux, respectively (Cichon et al., 2019). Due to the role of ATP7A in copper import, genetic mutations in *ATP7A* cause the neurological condition Menkes disease, in which patients develop a severe copper deficiency leading to symptoms of neurodegeneration, developmental delays and accelerated mortality (Hodgkinson et al., 2015). In contrast, a mutation in the *ATP7B* gene results in Wilson's disease, which causes copper accumulation and toxicity in the brain and liver as ATP7B is crucial for copper transport from the liver into bile (Czlonkowska et al., 2018; Mercer et al., 2017). Disturbances in copper homeostasis have also been observed in a variety of neurodegenerative diseases, including Alzheimer's disease (Li et al., 2012), Parkinson's disease (Bisaglia and Bubacco, 2020) and amyotrophic lateral sclerosis (Tokuda and Furukawa, 2016), as well as in patients with cardiac disease (de Romana et al., 2011). Thus, copper homeostasis is essential for human health as both deficiency and excess of copper result in significant pathology and are observed in multiple diseases.

### Copper enhances host immunity

Mammalian immune responses rely heavily on sufficient copper levels. Immune cells use copper-dependent mechanisms to activate protective immunity and destroy invading pathogens (Samanovic et al., 2012), with some studies indicating that dysregulation of copper levels leads to poor immune function, as was observed in patients with COVID-19 (Calder, 2020) and cancer (Denoyer et al., 2015). For example, dietary copper restriction during perinatal development in mice led to systemic copper deficiency, resulting in anaemia, reduced immune enzyme activity, enlarged spleens and small thymuses (Osredkar, 2011). The impaired immune response positively correlated with the severity and duration of copper deficiency (Windhauser et al., 1991). Similar leukocyte proliferation defects have been observed in humans with low copper levels, which result in a range of haematological problems including anaemia, neutropenia, leukopenia, thrombocytopenia and abnormalities in the bone marrow, including a reduction in granulocyte precursors (Halfdanarson et al., 2008). Copper deficiency also appears to affect myeloid cell function, as macrophages from rats fed a copper-deficient diet phagocytosed fewer yeast cells (Munoz et al., 2007).

Copper also boosts extracellular mechanisms of antimicrobial defence by enhancing the activity of antimicrobial peptides (AMPs). AMPs can be generated by a variety of cells, such as macrophages, epithelial cells, neutrophils and lymphocytes (Izadpanah and Gallo, 2005). Certain AMPs possess the amino-terminal copper- and nickel-binding (ATCUN) motif in their amino-terminal region (Wende and Kulak, 2015). The Cu-ATCUN complex increases antibacterial activity by promoting mitochondrial production of reactive oxygen species (ROS) leading to oxidative and endoplasmic reticulum (ER) stress, in addition to necroptosis (see Glossary, Box 2) in immune cells (Lv et al., 2019).

### Copper-dependent mechanisms of fungal virulence

Copper is also essential for the growth and function of microorganisms, including fungi. Fungal cells use copper for cellular processes, including cell wall formation, energy production, oxidative stress defences and respiration (Li et al., 2019). The acquisition and consumption of copper in fungi are under the control of copper-sensitive transcription factors that mediate downstream expression of transporter proteins and metallothioniens (Box 2) as required (Raffa et al., 2019). As our understanding of copper-dependent mechanisms of fungal virulence and antifungal defence are best understood for the fungal pathogen *Cryptococcus neoformans*, we will primarily focus on this fungus in this section as an example.

**Fig. 1.**
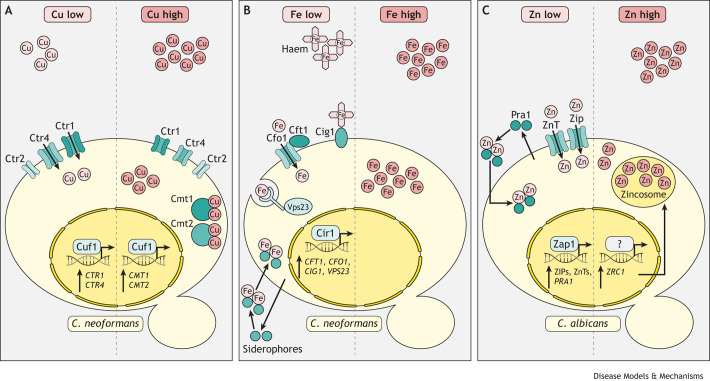
**Fungal metal-sensing systems and regulatory mechanisms.** (A) An overview of proteins and transcription factors used for responses to high and low environmental copper (Cu) in *Cryptococcus neoformans*. *C. neoformans* is unusual in that the same transcription factor, CnCuf1, regulates responses to both copper starvation (via upregulation of the copper transporters CnCtr1 and CnCtr4) and copper toxicity (via upregulation of the metallothioneins CnCmt1 and CnCmt2). CnCtr2 has more recently been linked to capsule formation to shield *C. neoformans* from innate immune recognition. (B) An overview of iron (Fe)-scavenging systems in *C. neoformans* under conditions of low iron availability, as would be found in the host. When free iron is limited, the transcription factor CnCir1 upregulates the iron permease CnCft1 and the ferroxidase CnCfo1, which form a complex to mediate iron import into the cell. CnCir1 also upregulates cell wall mannoprotein, CnCig1, to acquire iron from host haem. Furthermore, uptake of iron via endocytosis is mediated by the cytoplasmic protein CnVps23 and siderophores can be secreted to sequester extracellular iron. (C) Overview of zinc (Zn) uptake and storage systems in *Candida albicans*. When zinc is readily available, *C. albicans* stores zinc within intracellular zincosomes, the formation of which is controlled by CaZrc1. When zinc is limited in *C. albicans*, the transcription factor Zap1 upregulates the expression of CaPra1 to acquire this metal extracellularly and of the Zip and ZnT zinc transporter protein family.

Although many fungi use distinct transcription factors for sensing high and low concentrations of metals, such as copper, *C. neoformans* is unusual in that the same transcription factor regulates responses to both high and low copper concentrations (Ding et al., 2011) (Fig. 1A). *C. neoformans* (Cn) copper-responsive factor gene (CnCuf1) is the transcription factor that regulates the expression of genes encoding proteins required to adapt to copper deficiency (Ding et al., 2011). This includes copper transporters 1 and 4 (CnCtr1 and CnCtr4) to import copper, as well as proteins needed to protect against copper toxicity, such as copper-detoxifying metallothioneins 1 and 2 (CnCmt1 and CnCmt2) (Ding et al., 2011; Waterman et al., 2007; Sun et al., 2014). CnCuf1 contains a copper-binding motif at the carboxy-terminal region that is also found in other fungal copper-sensing transcription factors, and this transcription factor is essential for the pathogenicity of this fungus specifically in the brains of both mice and humans (see below) (Waterman et al., 2007).

Similar copper sensing systems have been described for other fungal pathogens. Like *C. neoformans*, *Histoplasma capsulatum* can infect macrophages and modify the phagosomal environment for intracellular growth, either by inhibiting phagolysosomal fusion or by buffering the acidic pH within the phagosome to maintain neutral growth conditions (Strasser et al., 1999; De Leon-Rodriguez et al., 2018). In conditions of low environmental copper, the transcription factor *H. capsulatum* (Hc)Mac1 upregulates the expression of HcCtr3 (Shen et al., 2018), a copper importer that is functionally similar to *C. neoformans* CnCtr4. *H. capsulatum* also encodes two other putative copper transporters, HcCtr1 (which is structurally related to *C. neoformans* CnCtr1) and HcCtr2; however, these do not appear to functionally compensate for HcCtr3 when fungal cells are copper starved and hence their specific functions are not well understood (Moraes et al., 2023; Shen et al., 2018). The mechanisms of copper detoxification in *H. capsulatum* have not been clearly defined.

The ability of fungi to sense and respond to host copper levels is closely related to their virulence and development of organ-specific responses (Gerwien et al., 2018). In mouse models of *C. neoformans* infection, a striking difference in copper regulation has been noted between the lung and brain. In the lung, *C. neoformans* highly expresses *CnCMT1* and *CnCMT2*, indicating that there is a high concentration of copper in this tissue (Sun et al., 2014). As such, *C. neoformans* mutant strains lacking expression of *CnCMT1* and *CnCMT2* are unable to establish pulmonary infection and are quickly cleared by immune cells (Sun et al., 2014; Ding et al., 2013), likely owing to their inability to detoxify high copper concentrations. In contrast to the lung, copper levels are much lower in the brain, resulting in downregulation of CnCmt1 and CnCmt2 and CnCuf1-dependent upregulation of CnCtr1 and CnCtr4 to enhance copper uptake (Sun et al., 2014). Indeed, a *CnCTR4*-deficient mutant of *C. neoformans* is unable to establish infection in the brain but, interestingly, this mutant is hypervirulent in models of lung infection (Sun et al., 2014), presumably due to the enhanced resistance against copper toxicity. Even within the brain, there are differences in *C. neoformans* copper starvation responses that depend on the micro-niche that the fungus inhabits. In our recent preprint, we found that although the fungus upregulates CnCtr4 in the extracellular space and within inflammatory macrophages in the brain, this does not occur within tissue-resident microglia (Mohamed et al., 2022 preprint). In *H. capsulatum*, expression of HcCtr3 is low when internalized within macrophages, indicating that this fungus may acquire sufficient copper within these host cells and increased copper uptake is not required (Shen et al., 2018). These results indicate that the relationship between copper homeostasis and virulence might vary depending on the host organs, tissue micro-niches and cell types infected.

This ability to adapt to differing levels of host copper is important as several classic *C. neoformans* virulence factors use or depend on copper. For example, the copper-dependent enzyme laccase is responsible for the production of melanin, which shields the fungus against oxidative stresses within the host (Janusz et al., 2020). *C. neoformans* also secretes a capsule to ‘hide’ from innate immune recognition and copper helps promote capsule formation by regulating the expression of *CnCTR2*, a gene that shares homology with previously identified fungal copper transporters (Chun and Madhani, 2010). Furthermore, copper is involved in promoting iron uptake, which, in turn, fuels other aspects of virulence (discussed in detail in the next section) (Waterman et al., 2007). As fungi rely on copper not only for growth, but also for specific virulence factors, the host has developed mechanisms to manipulate copper levels as an anti-fungal strategy.

### Copper manipulation as an anti-fungal strategy

**Fig. 2.**
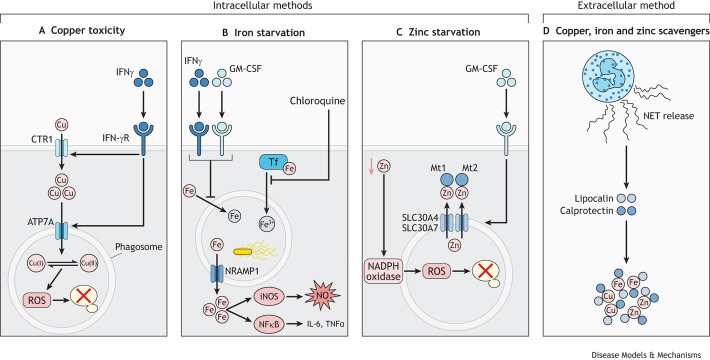
**Metal-dependent mechanisms of intracellular and extracellular killing by immune cells.** (A-C) Inflammatory cytokines, such as IFNγ and GM-CSF can initiate mechanisms that restrict metals from intracellular fungi and phagosomes. (A) During bacterial infection, IFNγ has been shown to drive the pumping of copper (Cu) into phagosomes to cause toxicity, which occurs via the upregulation of the mammalian copper importers CTR1 and ATP7A. High copper levels also contribute to the production of reactive oxygen species (ROS) via NADPH oxidase, which further enhances microbial killing. Conversely, IFNγ limits copper from intracellular fungi via an unknown pathway, causing fungi to upregulate genes involved with copper starvation (e.g. HcCtr3 in *Histoplasma capsulatum*). IFN-γR, IFNγ receptor. (B) Iron (Fe) in macrophages has been shown to be limited from intracellular pathogens following stimulation with IFNγ and GM-CSF. Chloroquine is a drug that limits intracellular fungal growth, in part by preventing the release of iron from transferrin. Extrusion of iron from the phagosome into the cytoplasm via NRAMP1 helps support inflammatory responses, including the expression of iNOS, leading to the production of nitric oxide (NO), and pro-inflammatory cytokines such as IL-6 and TNFα via NFκB. (C) GM-CSF activation in macrophages upregulates the zinc (Zn) exporters SLC30A4 and SLC30A7, which remove zinc from the phagosomes to starve the residing fungi and move it into the cytoplasm, where it is subsequently chelated by metallothioneins Mt1 and Mt2. Zinc limitation in the phagosome and cytoplasm supports ROS production to support fungal killing. (D) Neutrophils may also participate in limiting copper, iron and zinc from larger, extracellular fungi via calprotectin and lipocalin, a key component of neutrophil extracellular traps (NETs).

Both limiting copper and deploying toxic concentrations of copper may be used in host anti-fungal defence. For example, human macrophages can pump copper ions into microbe-containing phagosomes using ATP7A, which, in turn, boosts the production of ROS to impair the function of various fungal macromolecules, including proteins, lipids and DNA (Fig. 2A) (Hodgkinson and Petris, 2012). Copper ions can both receive and donate an electron while transitioning between the Cu(I) and Cu(II) oxidation states, which facilitates the generation of hydroxyl radicals through the Fenton and Haber–Weiss reactions (Liochev and Fridovich, 2002). Fungi may attempt to defend themselves against copper-supported generation of ROS. For example, *Aspergillus fumigatus* expresses the transcription factor *A. fumigatus* (Af)AceA, which promotes resistance to ROS and copper within the host. Deletion of *AfACE2* renders the fungus less virulent in mouse models of infection and more sensitive to copper when fungi were cultured alone, which could be overcome by inhibiting host production of ROS or enhancing export of copper by the fungus (Wiemann et al., 2017). These mechanisms by which copper boosts antimicrobial function of macrophages may also be modulated by inflammatory cytokines. For example, IFNγ (or IFNG) stimulation of mouse macrophages increases the expression of CTR1 by these cells, which enables greater intake of copper into the cell and subsequent movement of ATP7A to the phagolysosomal membrane (White et al., 2009). This movement of mammalian copper importers is required to drive an influx of copper into the phagosome, which boosts killing of phagocytosed bacteria via copper poisoning (White et al., 2009).

Conversely, as sufficient copper is needed for fungal growth and virulence, the host can also use strategies to starve fungi of copper. Contrary to the studies above (Shen et al., 2018), IFNγ has also been shown to restrict copper from phagosomes containing fungal pathogens. For instance, when macrophages were pre-activated with the cytokine IFNγ, HcCtr3 expression in *H. capsulatum* increased within the phagosome, which indicates copper starvation (Shen et al., 2018). Importantly, IFNγ can boost macrophage-mediated killing of *H. capsulatum*, but only of *HcCTR3*-deficient strains (Shen et al., 2018). We observed similar IFNγ-dependent copper restriction within brain macrophages during *C. neoformans* infection in our recent preprint (Mohamed et al., 2022 preprint). Although IFNγ can mediate copper restriction within host cells, the mechanism by which this occurs remains elusive (Fig. 2A). Despite this, it is apparent that fungi may counteract these strategies by upregulating the expression of copper importers to boost their access to these restricted micronutrients. It also remains unclear how macrophages make decisions about whether to deploy copper toxicity or starvation as strategies for microbial killing, but it is likely related to the pattern recognition receptors (PRRs) engaged (Box 1), and the cytokine-mediated activation and integration of downstream signalling pathways. Careful future studies are required to link innate fungal recognition with metal-dependent regulation of immune mechanisms.

## Iron

Iron is the most abundant metal in all organisms, playing critical roles in oxygen transport, DNA synthesis and the electron transport chain (Gurzau et al., 2003). In mammals, iron uptake primarily occurs through transferrin receptor 1 (TFRC) and divalent metal-ion transporter 1 (SLC11A2), whereas the release of iron from cells is facilitated by the iron export protein ferroportin (SLC40A1) (Anderson and Vulpe, 2009). The regulation of iron transport at the systemic level involves the liver-derived peptide hepcidin (HAMP), which controls iron release into the blood plasma by acting on ferroportin (Anderson and Vulpe, 2009). Similar to copper, iron concentrations must be tightly regulated as both excess and deficiency can cause a variety of medical conditions (Koleini et al., 2021). Iron deficiency results in anaemia, a condition associated with fatigue and mild cognitive impairment caused by poor oxygen delivery to the muscles and brain (Lal, 2020; Patterson et al., 2000; Agrawal et al., 2019). In contrast, hereditary hemochromatosis is a genetic disease caused by mutations in the gene *HFE*, which restricts iron movement into the bloodstream and is associated with the accumulation of iron in several organs, leading to restrictive cardiomyopathy, diastolic dysfunction and organ damage (Brissot et al., 2008).

### Iron homeostasis is required for host immune function

Imbalances in iron impair host immune responses and increase susceptibility to various invasive fungal infections. Liver transplant patients with excess iron have an increased risk of developing cryptococcosis, candidiasis, aspergillosis and mucormycosis, which suggests that these infections have the capability to exploit host iron as a vital factor in their virulence, as discussed below (Alexander et al., 2006). In terms of host immune responses, administering excess iron intracerebrally to mice infected with *C. neoformans* leads to a decrease in the production of IL-12 and IFNγ, resulting in an elevated fungal burden in the brain, spleen, lung and liver (Barluzzi et al., 2002).

Conversely, iron is required for general immune cell function, as a lack of this metal causes significant defects in immune cell proliferation due to impaired cellular and DNA replication. Recent studies have demonstrated that anaemia in patients is associated with decreased numbers of CD4 T cells (Hill et al., 2020), as well as defects in B cell responses and the generation of antibodies in response to vaccination (Stoffel et al., 2020). Therefore, systemic levels of iron need to be properly balanced in the host to maintain proper immune system functions and general immunity.

### Iron-dependent mechanisms of fungal virulence

Fungi use iron for a variety of biological functions, including respiration, DNA synthesis and the production of lipids and amino acids (Philpott, 2006). As mentioned above, excess iron in the host can promote susceptibility to fungal pathogens that would normally struggle to access host iron. For example, *Rhizopus* species grow poorly in human serum, partly owing to their inability to sequester host iron, and addition of exogenous iron to serum reverses their growth defects (Artis et al., 1982). *Rhizopus* species cause the disease mucormycosis, an infection that affects the sinuses and rhino-orbital spaces of immunocompromised patients. One of the major risk factors for developing *Rhizopus* infections is diabetic ketoacidosis (Box 2), which is associated with elevated levels of free iron in the serum (Andrianaki et al., 2018). Furthermore, patients treated with drugs that affect iron homeostasis have increased susceptibility to *Rhizopus* infections, often with enhanced mortality (Boelaert et al., 1994). For example, the iron chelator deferoxamine treats iron toxicity in patients but acts as a siderophore (Box 2) for *Rhizopus*, promoting its virulence and growth (Boelaert et al., 1993). Due to these striking clinical associations between host iron status and susceptibility to mucormycosis, iron-associated pathways have been targeted therapeutically as a potential way to combat the infection. Now, other types of iron chelators, which do not provide siderophore-mediated uptake to the fungus, have been shown to improve survival when used adjunctively with antifungal drugs in mouse models of mucormycosis (Ibrahim et al., 2007). However, small clinical trials in humans have been inconclusive in determining the efficacy of this approach in the clinic (Spellberg et al., 2012).

Unlike *Rhizopus*, most fungal pathogens have high-affinity iron uptake systems (Fig. 1B). The majority of host iron is bound to host proteins, such as haem, and there are very low levels of free ‘available’ iron, hence access to free iron is heavily restricted. These fungal pathogens therefore rely on intricate networks of iron-dependent transcription factors, transporters and storage proteins, as well as siderophores to acquire this metal from the host (Fig. 1B) (Haas et al., 2008). For example, CnCir1 is a master transcription factor controlling *C. neoformans* sensing of environmental iron and controls downstream expression of the iron permease (Box 2) CnCft1 and the ferroxidase CnCfo1, which form a complex to mediate iron import into the cell. Mutants lacking *CnCIR1* have significantly reduced virulence in animal models, which has been linked to disrupted expression of several classic virulence traits, including capsule formation and melanin production (Jung and Do, 2013; Jung et al., 2006). As iron affects multiple cellular processes (e.g. cell wall and capsule formation), it can be difficult to determine how mutants with disrupted iron uptake systems are compromised specifically in virulence mechanisms, and how much of their phenotype is attributed to changes in cell morphology and gene expression caused by iron deficiency. *C. neoformans* may also obtain iron from host haem via a cell wall mannoprotein called CnCig1. However, mutants lacking *CnCIG1* had no reduction in virulence unless the strain also lacked other iron uptake proteins, such as CnCfo1. Finally, uptake of iron via endocytosis is mediated by the cytoplasmic protein CnVps23 in *C. neoformans*, and Δ*vps23* strains were found to be avirulent in mouse models of infection (Cadieux et al., 2013; Hu et al., 2013).

Whereas *C. neoformans* has several iron uptake strategies, other fungi have more restrictive systems. *A. fumigatus* is unable to directly sequester host iron (Haas, 2012) and instead largely relies on the use of siderophores (Hohl and Feldmesser, 2007). Triacetylfusarinine C is a secreted siderophore that captures extracellular iron, which is then stored intracellularly using additional siderophores, ferricrocin and hydroxyferricrocin, within the fungal hyphae and conidia (Box 2), respectively (Seifert et al., 2008). The production of these siderophores depends on the ornithine monooxygenase AfSidA. Consequently, *ΔsidA* mutants have a complete loss of virulence in murine models of aspergillosis (Hissen et al., 2005). Other regulators of the siderophore system in *A. fumigatus* are the transcription factors AfSreA and AfHapX. When siderophores are not needed, due to sufficient or excess environmental iron, AfSreA suppresses the expression of siderophores, whereas AfHapX drives their expression when iron is limited, in part by inhibiting AfSreA function (Schrettl et al., 2010). Deletion of AfHapX in *A. fumigatus* impairs virulence, demonstrating that the ability to upregulate siderophore production within the host is critical for fungal survival in these hostile growth conditions (Schrettl and Haas, 2011). This system of siderophore regulation has also been shown to mediate *A. fumigatus* virulence in conditions where host iron is more readily available. Microhaemorrhages, caused by surgical procedures during transplantation, may provide an additional iron source and boost the invasive potential of *Aspergillus* species (Hsu et al., 2018). Fungal infection was enhanced in mouse tracheal transplant grafts containing higher concentrations of iron, associated with progressive graft rejection (Hsu et al., 2018). This was exacerbated in mice with hemochromatosis or in mice treated with iron-containing solutions. When these mice were infected with strains lacking AfSreA, infection and invasion of the graft was significantly reduced, which was attributed to the poor growth of the mutant in high iron levels (Hsu et al., 2018).

### Iron manipulation as an anti-fungal strategy

Immune cells can manipulate intracellular iron concentrations to mediate starvation and killing of pathogens residing within phagosomes. As outlined above for copper, the activation of macrophages with pro-inflammatory cytokines, including IFNγ and granulocyte-macrophage colony-stimulating factor (GM-CSF or CSF2), may limit the availability of metals such as iron, copper and zinc in the phagosome for fungi and may be a mechanism by which these cytokines exert their protective effects (Fig. 2B) (Winters et al., 2010; Mohamed et al., 2022 preprint; Shen et al., 2018). Indeed, IFNγ and GM-CSF are both protective cytokines against *C. neoformans* and *H. capsulatum* infections. Drugs that mimic the effects of these cytokines on intracellular iron levels have been shown to have therapeutic effects in mouse models of histoplasmosis and paracoccidioidomycosis (Dias-Melicio et al., 2007). For example, drugs that prevent the release of iron from transferrin (Tf), such as chloroquine, or chelate intracellular iron can limit the intracellular growth of *H. capsulatum* within macrophage phagosomes (Newman et al., 1994).

The transporter NRAMP1 (or SLC11A1) is found within the membrane of late phagosomes (Gruenheid et al., 1997) where it extrudes iron into the cytoplasm to deplete the metal from microbes within phagosomes (Fig. 2B), and, accordingly, macrophages lacking NRAMP1 are unable to restrict growth of several intracellular bacteria, including *Salmonella* Typhimurium and *Mycobacterium tuberculosis* (Murdoch and Skaar, 2022; Cellier et al., 2007). Depletion of iron from the phagosome consequently leads to increased iron concentrations within the cytoplasm, which have been shown to support expression of iNOS (or NOS2) (Box 2), which generates toxic nitric oxide (NO; Box 2) (Weiss et al., 1994), an important molecule for killing intracellular fungi. Enhanced iron in the cytoplasm also promotes activation of the transcription factor NFκB (Box 2) (She et al., 2002), which controls expression of multiple pro-inflammatory cytokines including TNFα (or TNF), IL-6 and IL-12. Beyond intracellular iron, neutrophils can modulate extracellular iron concentrations through the release of iron-scavenging proteins, such as calprotectin and lipocalin, both key components of neutrophil extracellular traps (NETs), which kill several fungal pathogens, such as *Candida albicans* (Zhong et al., 2022) (Fig. 2D). Taken together with the studies that show reduced virulence of fungi with disrupted iron uptake, it is clear that a mechanistic understanding of how immune cells restrict iron from fungi and how these pathogens might subvert these mechanisms is warranted and represents an important avenue for future investigation.

## Zinc

Zinc plays a critical role in cellular function by acting as a cofactor for multiple transcription factors, enzymes and structural proteins (Franklin and Costello, 2009). Effective regulation of cellular zinc levels in mammals is achieved through the coordinated activity of zinc transporters and zinc-sensing molecules, such as metallothioneins and metal-responsive-element-binding transcription factor-1 (MTF-1) (Fukada et al., 2011). Zinc deficiency can lead to malformations of the brain, inflammatory skin lesions and cardiac failure (Kozlowski et al., 2009). Zinc accumulation, in contrast, may impede the proper functioning of the immune system (Chasapis et al., 2012).

### Zinc enhances host immune function

Zinc deficiency can have significant repercussions for immune function. Patients with zinc deficiency exhibit a range of symptoms, such as high nitrogen levels (hyperammonaemia), decreased activity of thymus-produced enzymes (e.g. thymulin) and a decline in lean body mass (Prasad, 2014). These defects, in turn, lead to impaired development and function of innate immune cells, including neutrophils, natural killer cells and macrophages (Maares and Haase, 2016). Healthy people on a low zinc diet were found to have decreased functional capacity of natural killer cells and reduced production of IFNγ (Maares and Haase, 2016). These findings indicate that even a mild zinc deficiency in humans can have adverse effects on immunological function.

### Zinc-dependent mechanisms of fungal virulence

Fungi require zinc for growth within the host, as this metal acts as a cofactor for several transcription factors and enzymes involved in fungal stress responses, such as superoxide dismutase (Box 2) and alcohol dehydrogenase (Jung, 2015). In this section, we primarily focus on *C. albicans* as it is the best-understood example of zinc regulation and uptake with regards to virulence and infection in fungi (Fig. 1C).

The transcription factor *C. albicans* (Ca)Zap1 controls the expression of many genes in response to changes in environmental zinc within *C. albicans* (Eide, 2006). Under low zinc conditions, CaZap1 maintains the expression of two protein families, the Zip and ZnT transporters, that regulate the zinc transportation system (Wilson et al., 2012). *C. albicans* can also sequester zinc from the extracellular environment by secreting ‘zincophore’ scavenger protein CaPra1 (Wilson, 2015). Uptake of excess zinc may be toxic; hence, fungi address this potential threat by efficiently sequestering zinc within vesicular stores called zincosomes, providing a secure zinc source that may be utilised when environmental conditions change and zinc becomes limited. In *C. albicans*, the formation of zincosomes is regulated by the protein CaZrc1 (Crawford et al., 2018).

Fungal zinc transporters have also been described beyond *C. albicans*. First discovered in the model yeast *Saccharomyces cerevisiae*, several homologues of the zinc transporters Zrt1 and Zrt2 in fungal pathogens have now been described, for example, in *C. albicans and Cryptococcus gattii* (Do et al., 2016). Zrt1 and Zrt2 are zinc transporters that function in a pH-dependent manner and are required for adaption to low zinc environments (Bensen et al., 2004). Zrt2 functions in acidic pH, such as that found within macrophage phagosomes. In *C. neoformans*, homologues of the Zrt proteins (CnZip1 and CnZip2) were found to also regulate responses to environmental zinc (Do et al., 2016).

The regulation of zinc levels by fungi is essential for their pathogenicity. Deficiency of the transcription factor CaZap1 and/or the zinc importers in *C. albicans* leads to fungal growth defects and a reduction in virulence (Wilson, 2015). For intracellular replicating fungi, such as *H. capsulatum*, the lack of the zinc transporter HcZrt2 impairs intracellular survival and results in avirulence in mouse models of infection (Dade et al., 2016). The Zrt homolog CnZip1 was also required for *C. neoformans* virulence in an inhalational animal model of the infection (Do et al., 2016). Furthermore, the extracellular scavenger CaPra1 may help establish mucosal *C. albicans* infections, as *pra1*Δ*/*Δ strains were less able to invade endothelial cells without the addition of exogenous zinc (Citiulo et al., 2012). However, *pra1*Δ*/*Δ strains are hypervirulent in mouse models of disseminated infection (Box 2) (Soloviev et al., 2011), indicating a complex role for *C. albicans* zinc scavenging that may exhibit organ-specific effects and be shaped by the microenvironment.

The uptake, maintenance and regulation of zinc in *C. albicans* also plays various roles in fungal morphological changes and biofilm formation (Jung, 2015). Mutants lacking transcription factors regulating zinc uptake are often reported to have defects in hyphal formation – an important morphological form for *C. albicans* virulence, as strains lacking this ability are mostly avirulent in mouse models of infection – and the expression of several proteases, adhesins and toxins are associated with hyphal growth (Netea et al., 2015). Environmental depletion of zinc leads to the formation of giant, spherical *C. albicans* yeasts called Goliath cells, which are hyper-adherent and have delayed formation of hyphae *in vitro* (Malavia et al., 2017). Zinc therefore controls multiple aspects of virulence for various fungal pathogens and may represent a promising target for future therapeutic strategies, although this will first require an in-depth understanding of how the host manipulates zinc levels for immunity and the mechanisms that regulate these processes.

### Zinc manipulation as an anti-fungal strategy

Limiting fungal access to zinc is another potential host defence mechanism. Like copper, zinc-dependent killing mechanisms can be modulated by inflammatory cytokines, such as GM-CSF (Subramanian Vignesh et al., 2013). For example, during *H. capsulatum* infection, GM-CSF activation of macrophages impeded fungal intracellular growth by mediating zinc starvation (Subramanian Vignesh et al., 2013) (Fig. 2C). Mechanistically, GM-CSF drove expression of zinc exporters (SLC30A4 and SLC30A7), that shuttled zinc out of phagosomes away from intracellular fungi, and also upregulated metallothionein proteins (Mt1 and Mt2) to sequester zinc in the cytoplasm and further limit access to the fungus (Subramanian Vignesh et al., 2013). In addition, this reorganisation of zinc-importing machinery and sequestering of zinc within the cytoplasm in GM-CSF-treated macrophages boosted the production of ROS via NADPH oxidase (Box 2), further improving the fungal-killing efficiency of these macrophages (Subramanian Vignesh et al., 2013).

The host can also limit the availability of extracellular zinc to invading microbes through the action of the protein calprotectin, a major component of NETs released by neutrophils, which may also help sequester copper in addition to zinc (Fig. 2D) (Gilston et al., 2016). Fungi may compete with calprotectin for access to zinc, which has also been observed for some pathogenic bacteria (Besold et al., 2018). Consequently, mice lacking calprotectin are significantly more susceptible to *C. albicans* infection (Urban et al., 2009). Zinc therefore represents a potential therapeutic axis for treatment of these fungal infections, as manipulating either host shuttling of zinc or fungal uptake of this metal can affect the clinical outcome of infection. Future studies will be needed to determine efficient strategies for safely manipulating zinc levels in patients and the clinical outcomes of this type of therapeutic intervention.

## Concluding remarks

Although it is clear that metals play integral roles in regulating antimicrobial immunity, our understanding remains in its infancy. Determination of how reorganization and shuffling of intracellular metals are modulated by inflammation and cytokine stimulation will be an important dimension for our future understanding of how pathogens compete for resources within the host and the subsequent impact on downstream immune responses. Fungal infections continue to cause unacceptable levels of morbidity and deaths of vulnerable patients. Immunocompromised patients, who are at most risk of invasive fungal infections, are set to increase in the next few years due to increased use of immunomodulating drugs for cancer treatment, as well as higher levels of immunosuppression in the general population caused by chronic viral infections and related disorders (Weisberg et al., 2021). Targeting metal-dependent mechanisms of fungal virulence and/or host antifungal immune responses may be a novel way to limit infection by pathogenic fungi. Studies that reveal how metals influence host-fungal interactions expand our understanding of immune system regulation generally and provide an exciting new avenue for therapeutic exploration that may improve clinical outcomes of life-threatening fungal infections.
